# Secure Patient Messaging in Ophthalmology: Retrospective Natural Language Processing Study

**DOI:** 10.2196/96026

**Published:** 2026-10-28

**Authors:** Jiyeong Kim, Zoha Zahid Fazal, Jibreel Obaid Rehman, Sophia Y Wang, Robert Chang, Eleni Linos, Yasir Sepah

**Affiliations:** 1 Center for Digital Health, Stanford Medicine, Palo Alto, CA, United States; 2 Department of Ophthalmology, Byers Eye Institute, Stanford Medicine, 2370 Watson Court, Palo Alto, CA, 94303, United States, 1 6173081056, 1 6173081056

**Keywords:** digital health, ocular complaints, AI model, protected information, telemedicine

## Abstract

**Background:**

The rapid rise of secure patient messaging has transformed ophthalmic communication, with text volumes now exceeding prepandemic levels across health care systems. Our study uses a natural language processing (NLP) model to explore patterns in over 30,000 secure messages and identify common ocular complaints and differences by patient demographics.

**Objective:**

The objective of this study is to characterize the clinical and administrative concerns communicated through secure ophthalmology messaging and to assess differences in message content across patient sociodemographic groups.

**Methods:**

We conducted a cross-sectional study of deidentified, patient-initiated secure messages sent between June 2014 and July 2024. Patients with ophthalmic conditions who initiated secure electronic health record (EHR) portal messages were included. Of the 52,388 extracted message threads, 30,390 (58%) patient medical advice request (PMAR) messages routed solely to the Department of Ophthalmology from 4817 unique patients were assessed after the exclusion of questionnaires, courtesy messages, clinician responses, and messages routed to other health care departments. Of these unique patients, 2673 (55.5%) were female, 2740 (56.9%) were aged 50 years or older, 2346 (48.7%) were White, 4392 (91.2%) were non-Hispanic, and 2774 (57.6%) were married. NLP and large language model (LLM)–assisted topic classification were used to categorize message content. The distribution of message topics and frequency of clinical concerns were assessed and stratified by age, sex, race, ethnicity, and marital status. Differences in message frequency by demographic subgroup were assessed using 2-proportion z-tests.

**Results:**

Following preprocessing and topic modeling, 14,284 (47%) of the 30,390 messages could be predefined in relation to a topic cluster and were included in the final analysis. Nearly half of all 14,284 predefined messages addressed administrative issues, including 4166 (29.2%) scheduling-related inquiries, 1352 (9.5%) pharmacy and medication refill requests, and 581 (4.1%) insurance document issues. Among clinical concerns, of the total 14,284 predefined messages, 2975 (20.8%) messages involved vision disturbances, 1241 (8.7%) involved glaucoma-related symptoms, 1074 (7.5%) involved tumor-related questions, and 1064 (7.4%) involved surgery-relevant concerns. Message content differed significantly by demographic characteristics. Non-White patients more frequently raised issues related to pharmacy refills, insurance, glaucoma, and disability documentation, whereas White patients more often reported surgical concerns. Older patients more frequently messaged about glaucoma, surgery, and tumor-related issues, while female patients more often reported complications and swelling or infection.

**Conclusions:**

Secure patient messages frequently include clinically relevant symptoms with potential triage implications and demonstrate demographic differences in care-seeking behavior. Systematic analysis of message content may support safer triage, improved workflow efficiency, and more equitable delivery of ophthalmic care.

## Introduction

The rapid rise of secure patient messaging has transformed ophthalmic communication, with text volumes now exceeding prepandemic levels across health care systems [1]. Although remote consultations are not new to ophthalmic care (in 1994, over 8000 calls were logged in an ophthalmic emergency department of a single health center [2], reflecting early forms of remote triage for urgent eye concerns), the integration of secure electronic messaging within electronic health records (EHRs) has enabled a shift from undocumented phone advice to traceable asynchronous care. Secure patient messaging currently bridges this gap by enabling prompt advice and triaging even with limited history and examination findings [2,3]. Although messages reflect the concerns of interest of patients, there is scarce literature on the range of content of patient inquiries for ophthalmology and how clinicians handle these inquiries digitally.

Understanding these messages can illuminate the patient concerns (eg, vision changes, postsurgery questions) that are often recorded but rarely analyzed systematically within EHR logs, which can potentially improve care delivery. Prior research shows that patient portal use varies by age, socioeconomic status, and race, raising concerns that digital health tools might inadvertently widen care gaps [2]. In ophthalmology, where certain populations face disproportionate disease burdens, such inequities can be particularly consequential for digital engagement. Our study, therefore, examines whether differences in message content and volume exist across demographic groups, providing insight into how patient messaging behaviors may reflect (or reinforce) existing disparities in access to care.

The emergence of innovative AI methodologies for natural language processing (NLP) has improved the feasibility of large-scale analysis of patient text message data [4]. Automation of initial clinical encounters using NLP models is now being widely tested using patient-generated messages available in online forums or secure digital health applications [5-7]. By using a similar methodology, our study explores patterns in over 30,000 secure messages to identify common ocular complaints and differences by patient demographics. The findings will provide insights into formulating AI tools for clinical assistance that can reduce the burden of clinical inboxes, prevent the alert fatigue faced by clinicians, and empower patient-centered care.

## Methods

### Data Source and Study Population

We identified individuals with ophthalmic disease using the International Classification of Diseases, 10th Revision (ICD-10), codes [8] listed in Table S1 in Multimedia Appendix 1. We extracted a total of 52,388 deidentified, secure messages exchanged through the patient portal of a large tertiary academic hospital, Stanford Health Care, over a period of 10 years (June 2014 to July 2024). We excluded 18,126 (34.6%) messages, including questionnaires, courtesy messages, and physician responses, and 3872 (7.4%) messages routed to nonophthalmology specialties, as shown in Figure S1 in Multimedia Appendix 1. This resulted in 30,390 (58%) patient medical advice requests (PMARs) extracted from the original database of 52,388 secure messages, which were routed to 13 ophthalmology clinics affiliated with Stanford Health Care in Northern California.

### Topic Modeling

To understand patients’ concerns and issues, we developed an NLP topic model using BERT (bidirectional encoder representations from transformers), as illustrated in Figure S1 in Multimedia Appendix 1. First, we preprocessed the raw message data by removing duplicates, special characters, less meaningful messages (eg, less than 50 characters, including “thank you”), and stopwords using CountVectorizer from the scikit-learn library. Second, we applied a widely used pretrained and distilled BERT model (all-miniLM-L6-v2) to convert each message to sentence-level embeddings, which was subsequently simplified by dimension reduction via Uniform Mapping and Approximation and Projection (UMAP). Then, we created a list of topics, applying zero-shot clustering by setting the topic similarity threshold of 0.82, generally considered a good score [9]. Using this threshold, approximately 16,106 (53%) of the 30,390 messages were not assigned to a defined topic cluster, reflecting conservative topic assignment to maximize semantic coherence, which left 14,284 (47%) messages for topic characterization and subsequent analyses. Messages that did not meet the similarity threshold were excluded from downstream topic-specific analyses. Each topic was plotted in the visualization to show the topics by size for quantification and location for relationships between topics.

### Interpretation of Patients’ Issues

Each topic was characterized by its 15 most representative keywords. Two investigators (JK and ZZF) manually reviewed the topics and keywords and grouped similar topics into a larger topic. For enhanced interpretability, we applied a large language model (LLM), OpenAI o4-mini (OpenAI), to elaborate on patients’ issues in the HIPAA (Health Insurance Portability and Accountability Act)–compliant analytics environment. As shown in Figure 1, multiple prompt-engineering techniques were used, including role prompting (eg, “you are a professional ophthalmologist in the hospital”), directive commanding (eg, “help interpreting the topic using keywords”), and expertise emulation (eg, “I am also an ophthalmologist, and I will use your response”).

**Figure 1.**
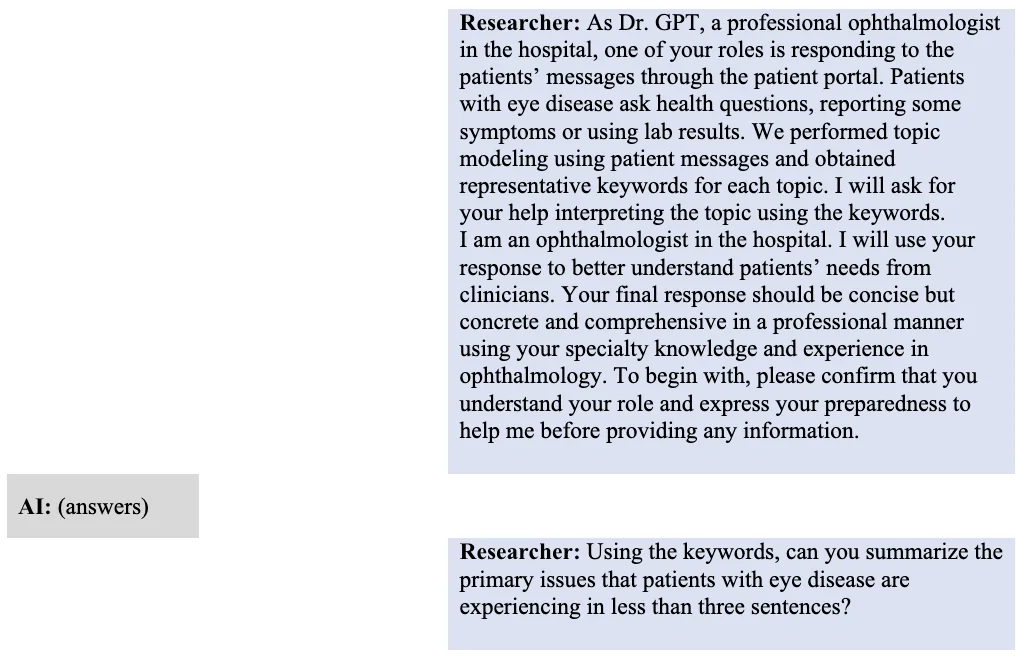
Engineered prompts used to interpret the key topics from natural language processing. GPT: generative pretrained transformer.

### Ocular Concerns by Sociodemographic Group

To identify patient groups who would need additional support for care, we computed the rate difference in topics addressed through patient messages by sociodemographic characteristics: age (under 50 years vs 50 years and older), sex (female vs male), race (White vs non-White, including Asian, Black/African American, Native American, Pacific Islander, other, and unknown), ethnicity (Hispanic vs non-Hispanic), and marital status (married vs unmarried, including single, divorced, separated, widowed, life partner, and other). The Rate_A_ was calculated such that the number of patients in subgroup A who had sent messages on topic X was divided by the total number of patients in subgroup A as computed below.


$${\text{Rate}}_{A}=\frac{\text{Number of patients in subgroup A who messaged on topic X}}{\text{Total number of patients in subgroup A}}$$

Because individual patients might have sent messages that were then grouped under multiple topics, these topic-specific rates were calculated independently and were therefore not expected to sum to 100% within a demographic subgroup. The statistical significance of the rate difference was determined by a 2-proportional z-test when *P*<.05. To visualize the relationships of the age and race categories with the patient issues, we plotted a Sankey diagram using detailed age (18 to <35 years, 35 to <50 years, 50 to <65 years, 65 to <75 years, and 75 years and older) and race (Asian, Black, Native American/Pacific Islander, other, and White) categories. We used Python 3.10 in Colab Enterprise (Google).

### Ethical Considerations

This study was approved by the Stanford University Institutional Review Board (IRB number 71157). The requirement for informed consent was waived because the study involved a retrospective analysis of deidentified patient data, posed minimal risk to participants, and could not practicably be conducted with individual consent. No direct patient contact occurred. All data were analyzed in a secure, HIPAA-compliant environment, and no personally identifiable information was included in the analysis or presented in this manuscript. Our research follows the Strengthening the Reporting of Observational Studies in Epidemiology (STROBE) guidelines.

## Results

A total of 30,390 secure portal messages from 4817 individuals with ophthalmic disease sent over a 10-year period were analyzed. The available cohort demographics were diverse: of these individuals, 2673 (55.5%) were female, 2740 (56.9%) were aged 50 years or older, 2471 (51.3%) self-identified as non-White, 4392 (91.2%) reported being of Non-Hispanic ethnicity, and 2774 (57.6%) reporting married status, as summarized in Table 1. This demographic snapshot highlights broad participation across sex, age, race/ethnicity, and marital status, although younger patients and Hispanic patients contributed proportionally fewer messages.

**Table 1.** Baseline demographics of patients messaging for ophthalmic concerns on the patient portal.

Patient demographics	Participants (4817), n (%)
Age (years)	
18 to <35	816 (16.9)
35 to <50	1261 (26.2)
50 to <65	1176 (24.4)
65 to <75	829 (17.2)
75 and older	735 (15.3)
Sex	
Female	2673 (55.5)
Male	2144 (44.5)
Race	
White/Caucasian	2346 (48.7)
Asian	1318 (27.4)
Other	757 (15.7)
Unknown	247 (5.1)
Black/African American	114 (2.4)
Native American	19 (0.4)
Pacific Islander	16 (0.3)
Ethnicity	
Non-Hispanic	4392 (91.2)
Hispanic/Latino	425 (8.8)
Marital status	
Married	2774 (57.6)
Unmarried	2043 (42.4)

Following preprocessing and topic modeling, 14,284 (47%) of the 30,390 included messages met the criteria for assignment to a topic cluster, so only those messages were analyzed, as illustrated in Figure 2.

**Figure 2.**
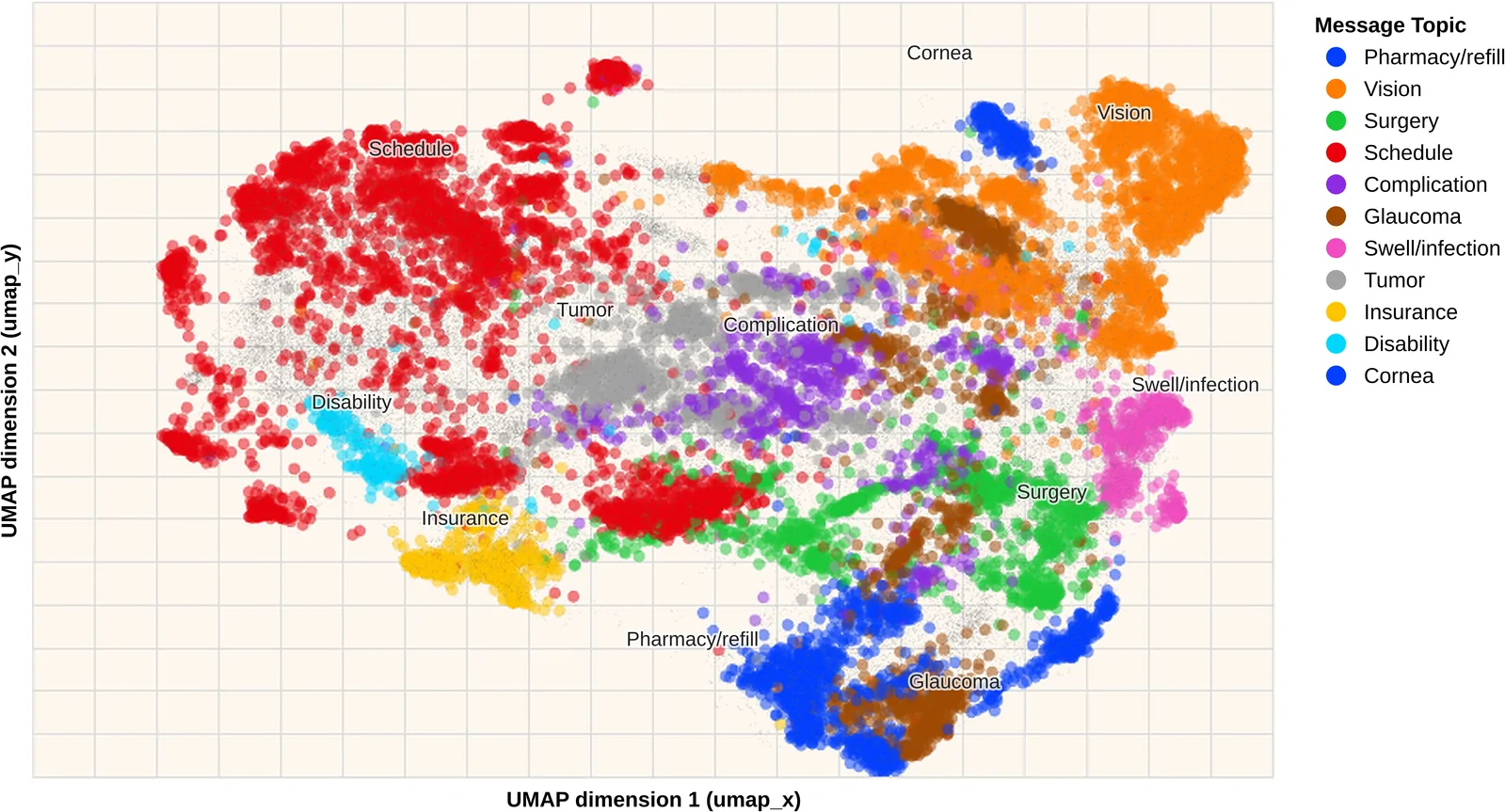
Low-dimensional representation of patient messages sent to Stanford Health Care’s Department of Ophthalmology, colored by topic. UMAP: Uniform Manifold Approximation and Projection.

Our model helped us classify ophthalmic message topics by administrative vs clinical issues, as shown in Table S2 in Multimedia Appendix 1. Although we only used PMARs, administrative concerns accounted for nearly half of all classified messages, as summarized in Table S3 in Multimedia Appendix 1. Scheduling-related inquiries were the most common message type of the predefined 14,284 message threads, accounting for 4166 (29.2%) messages, while medication refill– and pharmacy-related requests accounted for 1352 (9.5%) messages. Among clinical concerns, vision disturbances, including blurred vision, floaters, and flashes, comprised the most common topic, accounting for 2975 (20.8%) messages. Glaucoma-related questions comprised 1241 (8.7%) messages, while inquiries regarding imaging or tumor evaluation accounted for 1074 (7.5%) messages. Surgery-related concerns, including preoperative and postoperative issues, represented 1064 (7.4%) messages.

Figure 3 illustrates the distribution of patient message topics across age and race groups, where the width of each flow is proportional to the number of patients represented. Clinical concerns predominated over administrative issues in all demographic subgroups, with vision-related messages representing the largest clinical category and scheduling requests the most common administrative topic. Patients aged 50 years and older contributed a substantially greater proportion of messages than those aged less than 50 years, while White patients contributed a greater absolute number of messages than non-White patients. Despite these differences in message volume, the overall distribution of administrative and clinical concerns was similar across demographic groups, with vision, glaucoma, surgery, and scheduling consistently representing the most frequent topics.

**Figure 3.**
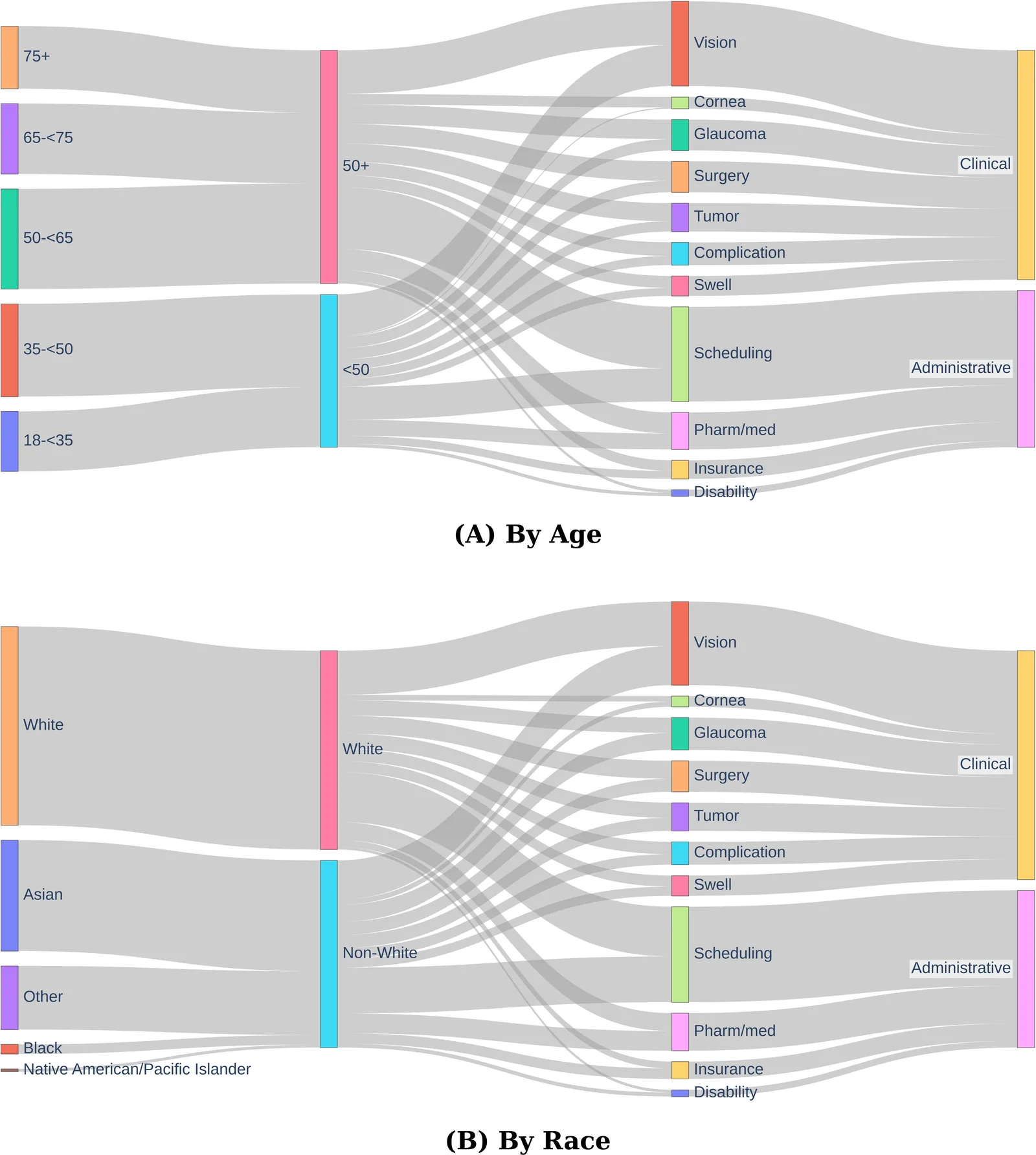
Message topics by size and demographic characteristics.

Differences by patient demographics were also noted, as shown in Figure 4. Patients aged 50 years and older contributed proportionally more clinical messages than younger patients. They more often asked about glaucoma (*P*<.05), tumor imaging (*P*<.05), surgery (*P*<.05), and cornea-related issues (*P*<.005) (Figure 4A). Non-White patients more frequently messaged about pharmacy refills (*P*=.03), glaucoma (*P*=.01), insurance (*P*=.004), and disability documentation (*P*=.02) than White patients, while White patients were more likely to inquire about surgery (*P*<.0001) (Figure 4B). Non-Hispanic patients sent more messages about vision concerns (*P*<.0001) and cornea issues (*P*=.017) compared to Hispanic patients (Figure 4C). Female patients more often reported complications (*P*=.02) and swelling/infection (*P*=.001) compared to males (Figure 4D). Unmarried patients more frequently sought advice on vision (*P*<.001) and disability (*P*<.001) issues, whereas married patients more often messaged about swelling/infection (*P*=.04), glaucoma (*P*=.03), and cornea concerns (*P*=.03) (Figure 4E).

**Figure 4.**
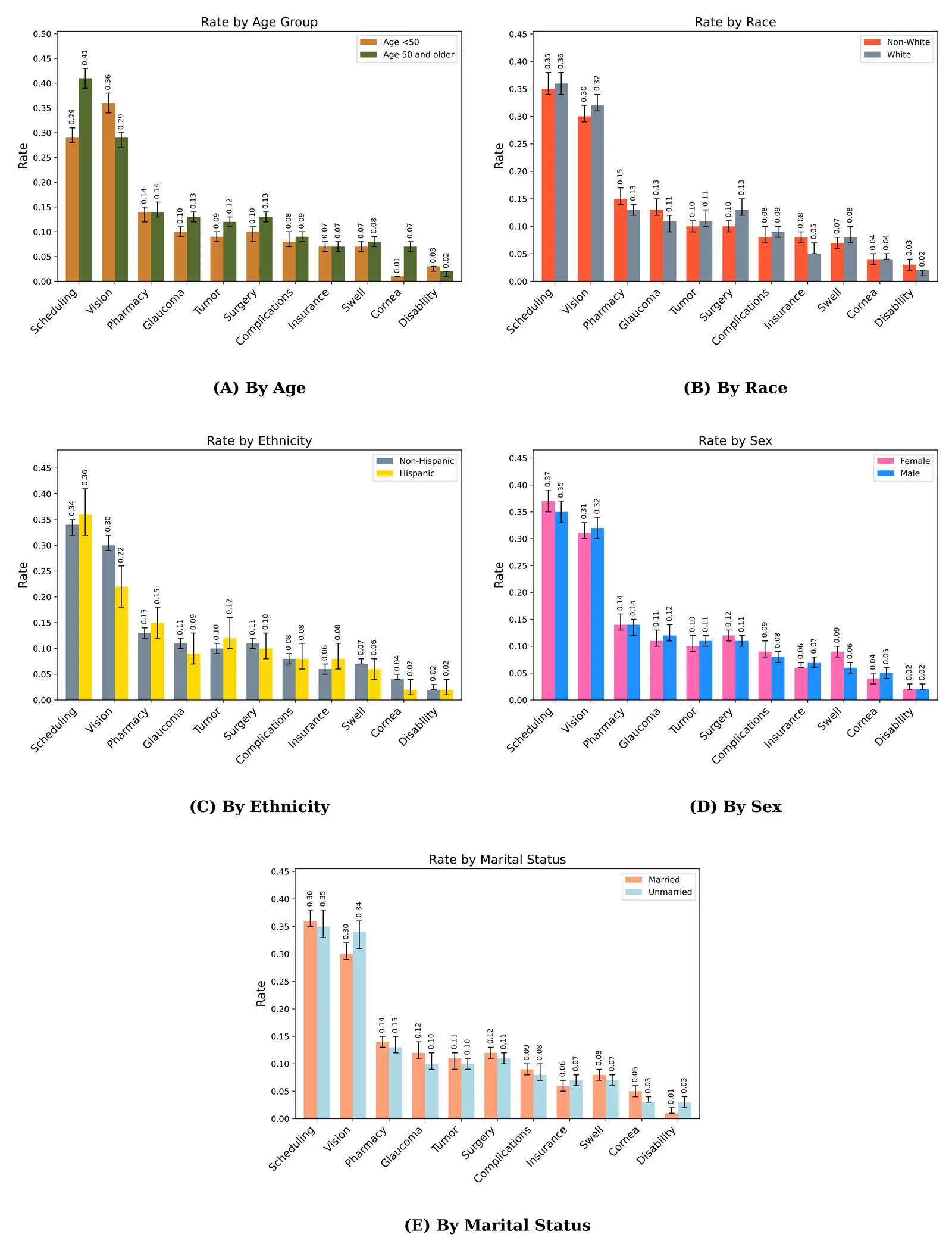
Message rates and topics stratified by age (A), race (B), ethnicity (C), sex (D), and marital status (E). For each demographic subgroup, the rate represents the proportion of patients who sent at least one message on a given topic (number of patients with ≥1 message on topic X divided by the total number of patients in that subgroup). Because individual patients may contribute to multiple message topics, rates are independent and do not sum to 1.

## Discussion

### Principal Findings

Our findings should be interpreted in the context of prior studies examining ophthalmology-related questions on public online platforms. Previous analyses of online Reddit and EyeTriage forum posts demonstrated that patients frequently seek information regarding ocular symptoms, treatment options, and urgent eye concerns outside traditional clinical settings [10,11]. Unlike these studies, however, our study evaluated secure patient portal messages exchanged directly between patients and their clinical care teams within the EHR system. As a result, our dataset captures real-world clinical and administrative concerns linked to ongoing care, allowing examination of demographic differences in messaging patterns across a large ophthalmology population. To our knowledge, this study represents one of the largest analyses of secure patient-provider ophthalmology messaging within an integrated EHR system and provides insights into patient communication patterns, topic distributions, and demographic differences in portal-based ophthalmic care. We found that scheduling requests and vision-related concerns were the most frequent message topics, alongside glaucoma and surgical questions, also highlighting subtle but important disparities in who uses these digital services. These insights provide a roadmap for improving digital ophthalmic care delivery. This analysis of over 30,000 ophthalmology-related messages received over a 10-year span on the patient portal reveals clear demographic patterns, distinct thematic trends in patient concerns, and actionable opportunities for AI-assisted triage. By linking large-scale NLP topic modeling with sociodemographic analysis, our findings highlight both the promise and the challenges of integrating digital messaging into ophthalmic care. Knowing the most common message topics (and which topics often signal urgent issues) allows the development of AI triage algorithms that flag critical ocular symptoms for prompt attention. Our data could inform training of an NLP system to distinguish routine questions from red-flag symptoms, such as sudden vision loss, that require a same-day response.

More than half of the message senders were female (2673/4817, 55.5%), and more than half were aged 50 years or older (2740/4817, 56.9%), consistent with the demographic profile of many ophthalmic conditions [12,13]. The predominance of women aligns with prior digital health literature suggesting higher female engagement with patient portals, possibly due to greater health-seeking behavior, higher prevalence of certain eye conditions, or the convenience of remote messaging for those with mobility or caregiving responsibilities [14]. Racial and ethnic distributions reveal potential access dynamics: although White patients comprised the largest racial group, non-White patients accounted for a slightly greater proportion of total message senders than seen in traditional in person visit patterns [12]. Notably, non-White patients more frequently messaged about glaucoma, pharmacy refills, insurance, and disability, while White patients disproportionately messaged about surgical issues, patterns that likely reflect known differences in disease burden, insurance complexity, and procedure uptake [15].

The finding that non-White patients raised more administrative and chronic disease management concerns underscores existing disparities in health care navigation and access [15]. Barriers such as insurance authorization complexity and pharmacy access may disproportionately affect these groups, suggesting that portal-based AI tools could be used to streamline such processes. Conversely, the higher rate of surgery-related messages from White patients may reflect greater access to elective or nonurgent procedures, emphasizing the need to monitor whether digital tools inadvertently reinforce existing inequities [13].

While older patients were not underrepresented in portal use, they contributed proportionally more clinical queries (particularly on glaucoma, surgery, and tumor imaging) than younger patients, highlighting age-related differences in how the portal is used. This likely reflects both the higher prevalence of chronic, vision-threatening conditions such as glaucoma, cataract, and ocular tumors in older adults and their greater need for perioperative communication given the frequency of surgery in this age group. In contrast, younger patients may rely on in person visits or alternate communication methods for routine concerns, resulting in fewer portal-based clinical queries. These findings align with emerging evidence that older adults increasingly engage with digital health technologies. Recent national survey data demonstrated substantial use of personal EHRs among older adults, particularly among those with greater health care needs [16,17]. Our findings extend this literature by suggesting that older adults not only access patient portals but also use them as a clinically meaningful communication channel for managing chronic ophthalmic conditions and treatment-related concerns.

While nearly half of all messages involved administrative matters such as scheduling and refills, clinical issues such as vision disturbances, glaucoma, and surgery-related concerns were highly represented. The strong presence of chronic disease topics such as glaucoma (particularly among older and non-White patients) reinforces the potential of patient messaging as an early touchpoint for ongoing disease monitoring, adherence reinforcement, and symptom triage [18,19].

Given that many high-volume, low-complexity message types (eg, scheduling, refills, insurance verification) could be automated, AI-driven triage systems could significantly reduce clinician burden [2,4]. Our NLP approach successfully identified topical categories, suggesting feasibility for real-time routing of urgent messages (eg, acute vision changes) to clinicians while directing routine queries to automated workflows [5,7]. Previous studies show that LLMs can approach ophthalmology trainee accuracy in triage and diagnosis, but accuracy, bias mitigation, and safety require further validation in live settings [20]. The variation in topics by demographic group also underscores the importance of training and testing AI systems on diverse datasets to avoid perpetuating disparities [21].

An AI-enabled portal triage system could flag urgent ophthalmic complaints for expedited review, automate routine administrative responses, and support multilingual or literacy-sensitive messaging for patients at risk of being underserved [22-24]. Integration with EHR alerts, automated follow-up prompts, and links to teleophthalmology services could further extend the reach of such systems, especially in areas with limited specialist access [19,25].

This study was conducted within a large academic health care system in Northern California and may therefore reflect the demographic composition, referral patterns, and digital health infrastructure of this region. Patients using secure messaging may differ from those receiving care in community-based, rural, or underserved health care settings. Furthermore, disparities in internet access, digital literacy, language proficiency, socioeconomic status, and patient portal enrollment may have influenced messaging behavior and contributed to observed demographic differences. These factors should be considered when generalizing the findings to broader populations and when developing equitable AI-assisted triage systems. Despite these limitations, the large sample and clear patterns support the main conclusions of this study.

Additionally, nearly half of the messages were not assigned to predefined topic clusters due to the conservative similarity threshold used in zero-shot clustering. While this approach improves topic specificity, it may limit completeness. Future work should explore lower similarity thresholds or supervised classification methods to improve coverage and classification accuracy. Similar analyses could benefit any field where patient portals are used, and our approach demonstrates how mining patient messages can identify unmet needs and system inefficiencies. The predominance of administrative and nonacute clinical messages, such as medication refills, dry eye symptoms, and postsurgical follow-ups, suggests that many patient concerns may be suitable for remote management or asynchronous triage, although message urgency was not formally analyzed in this study. While our analysis characterizes the content and demographic patterns of patient messages, we were unable to determine how each message was triaged in practice, whether it prompted an appointment, a same-day urgent visit, or an asynchronous reply. Because message outcomes were not linked to clinical scheduling or chart data, the urgency of individual threads could not be independently verified. This limitation should be considered when interpreting the potential of NLP tools for real-world triage, as their clinical utility ultimately depends on validated outcome mapping.

The study period spanned a decade from 2014 to 2024, encompassing substantial changes in health care delivery, patient portal adoption, and digital health technology, including the COVID-19 pandemic, which produced a marked and durable increase in secure messaging volume across health systems [1,26]. Message volume, topic distribution, and portal engagement may therefore have varied across prepandemic, pandemic, and postpandemic periods. Our topic model was derived from the pooled decade-long corpus to maximize semantic coherence and cluster stability; period-stratified rederivation of topics, which would be required to compare content across eras without cluster contamination, was outside the scope of this characterization and represents an important direction for future work. Examining how message content and the demographic composition of senders shifted across pandemic periods may further refine the design and ongoing calibration of AI-assisted triage tools, which are sensitive to temporal drift in input data. Building on these findings, future work should examine whether portal use correlates with downstream outcomes such as appointment adherence, surgical uptake, or reduced emergency department visits [26,27]. Prospective evaluation of AI triage performance in real-world ophthalmology workflows will be critical. Furthermore, targeted interventions (such as insurance navigation support for non-White patients and early glaucoma education prompts for high-risk groups) could be tested to assess their impact on health equity and clinical outcomes.

### Conclusions

Secure patient messaging, enhanced by NLP, reveals meaningful patterns in ophthalmic concerns and disparities in care-seeking behaviors. Non-White patients more often raised issues related to insurance, pharmacy refills, and chronic disease management, while White patients focused more on surgical topics, underscoring opportunities for targeted interventions. The prevalence of high-volume, low-complexity queries supports integrating AI-assisted triage into patient portals to prioritize urgent complaints, automate routine responses, and improve multilingual, literacy-sensitive communication. Such systems have the potential to enhance efficiency, equity, and timely access to specialty care but must be validated for accuracy, safety, and bias mitigation before widespread implementation. These findings support the development of AI-driven triage systems using real-world patient-generated data in EHR portals. By aligning AI-driven triage with equity-focused design, patient portals can transform from passive communication tools into proactive, data-driven components of ophthalmic care delivery.

## Supplementary material

10.2196/96026 Multimedia Appendix 1 Supplementary tables and figure including ophthalmic International Classification of Diseases, 10th Revision, diagnostic codes used to identify patients, predefined message categorization, ophthalmic message topics classified by administrative versus clinical issues, and the workflow of patient message data extraction, processing, and analysis.

## Abbreviations

BERT: bidirectional encoder representations from transformers
EHR: electronic health record
HIPAA: Health Insurance Portability and Accountability Act
ICD-10: International Classification of Diseases, 10th Revision
IRB: institutional review board
LLM: large language model
NLP: natural language processing
PMAR: patient medical advice request
STROBE: Strengthening the Reporting of Observational Studies in Epidemiology

## References

[1] Holmgren AJ, Downing NL, Tang M, Sharp C, Longhurst C, Huckman RS (2022). Assessing the impact of the COVID-19 pandemic on clinician ambulatory electronic health record use. J Am Med Inform Assoc.

[2] Alsumait A, Deshmukh S, Wang C, Leffler CT (2025). Triage of patient messages sent to the eye clinic via the electronic medical record: a comparative study on AI and human triage performance. J Clin Med.

[3] Ittarat M, Cheungpasitporn W, Chansangpetch S Personalized care in eye health: exploring opportunities, challenges, and the road ahead for chatbots. J Pers Med.

[4] Kim J, Chen ML, Rezaei SJ (2024). Perspectives on artificial intelligence–generated responses to patient messages. JAMA Netw Open.

[5] Clay B, Bergman HI, Salim S, Pergola G, Shalhoub J, Davies AH (2025). Natural language processing techniques applied to the electronic health record in clinical research and practice - an introduction to methodologies. Comput Biol Med.

[6] Mermin-Bunnell K, Zhu Y, Hornback A (2023). Use of natural language processing of patient-initiated electronic health record messages to identify patients with COVID-19 infection. JAMA Netw Open.

[7] Adejumo P, Thangaraj PM, Dhingra LS (2024). Natural language processing of clinical documentation to assess functional status in patients with heart failure. JAMA Netw Open.

[8] ICD-10-CM for Ophthalmology. American Academy of Ophthalmology.

[9] Hossain B, Preum SM, Rabbi MF, Ara R, Ali ME (2025). Extracting symptoms of complex conditions from online discourse (subreddit to symptomatology): lexicon-based approach. JMIR Med Inform.

[10] Dave AD, Zhu D (2022). Ophthalmology inquiries on Reddit: what should physicians know?. Clin Ophthalmol.

[11] Mahjoub H, Prabhu AV, Sikder S (2020). What are ophthalmology patients asking online? An analysis of the eye triage subreddit. Clin Ophthalmol.

[12] Brant A, Kolomeyer N, Goldberg JL (2023). United States population disparities in ophthalmic care: blindness and visual impairment in the IRIS® Registry (Intelligent Research in Sight). Ophthalmology.

[13] Rasendran C, Tye G, Knusel K, Singh RP (2020). Demographic and socioeconomic differences in outpatient ophthalmology utilization in the United States. Am J Ophthalmol.

[14] Couture A, Birstler J (2023). The gender of the sender: assessing gender biases of greetings in patient portal messages. J Womens Health (Larchmt).

[15] Greig EC, Gonzalez-Colaso R, Nwanyanwu K (2023). Racial, ethnic, and socioeconomic disparities drive appointment no-show in patients with chronic eye disease. J Racial Ethn Health Disparities.

[16] Agrawal L, DaSouza RO, Mulgund P, Chaudhary P (2025). Frequency of electronic personal health record use in US older adults: cross-sectional study of a national survey. JMIR Aging.

[17] Cross DA, Levin Z, Raj M (2021). Patient portal use, perceptions of electronic health record value, and self-rated primary care quality among older adults: cross-sectional survey. J Med Internet Res.

[18] Channa R, Zafar SN, Canner JK, Haring RS, Schneider EB, Friedman DS (2016). Epidemiology of eye-related emergency department visits. JAMA Ophthalmol.

[19] Wedekind L, Sainani K, Pershing S (2016). Supply and perceived demand for teleophthalmology in triage and consultations in California emergency departments. JAMA Ophthalmol.

[20] Lyons RJ, Arepalli SR, Fromal O, Choi JD, Jain N (2024). Artificial intelligence chatbot performance in triage of ophthalmic conditions. Can J Ophthalmol.

[21] Bernstein IA, Zhang YV, Govil D (2023). Comparison of ophthalmologist and large language model chatbot responses to online patient eye care questions. JAMA Netw Open.

[22] Atta S, Omar M, Kaleem SZ, Waxman EL (2022). The use of mobile messaging for telecommunications with patients in ophthalmology: a systematic review. Telemed J E Health.

[23] Khan IA, Bashar MA, Tripathi A, Priyanka N (2024). The benefits and challenges of implementing teleophthalmology in low-resource settings: a systematic review. Cureus.

[24] Dolar-Szczasny J, Barańska A, Rejdak R (2023). Evaluating the efficacy of teleophthalmology in delivering ophthalmic care to underserved populations: a literature review. J Clin Med.

[25] Meshkin RS, Armstrong GW, Hall NE, Rossin EJ, Hymowitz MB, Lorch AC (2023). Effectiveness of a telemedicine program for triage and diagnosis of emergent ophthalmic conditions. Eye (Lond).

[26] Sanayei N, Albrecht MM, Martin DC (2022). Outcomes of a hybrid ophthalmology telemedicine model for outpatient eye care during COVID-19. JAMA Netw Open.

[27] Abbas K, Abdulrahman M, Abbas AF, Marwaha A, Jaques M, Gizicki R (2025). Patient and appointment characteristics associated with no-shows at a multicenter retina ophthalmology practice. Can J Ophthalmol.

