# Supplementary material for: Secure Patient Messaging in Ophthalmology: Retrospective Natural Language Processing Study
Source: JMIR Med Inform. 2026 Oct 28;14:e96026. doi: 10.2196/96026 (PMC13717337; doi:10.2196/96026)
Supplement: Supplementary file 1 [file medinform-v14-e96026-s001.docx]

| **Ophthalmic ICD-10 Codes** | |
| --- | --- |
| **Amblyopia** | H53.001, H53.002, H53.003, H53.009, H53.011, H53.012, H53.013, H53.019, H53.021, H53.022, H53.023, H53.029, H53.031, H53.032, H53.033, H53.039, H53.041, H53.042, H53.043, H53.049 |
| **Uveitis** | H20.011, H20.012, H20.013, H20.019, H20.021, H20.022, H20.023, H20.029, H20.031, H20.032, H20.033, H20.039, H20.041, H20.042, H20.043, H20.049, H20.10, H20.11, H20.12, H20.13, H20.19, H30.021, H30.022, H30.023, H30.029, H30.031, H30.032, H30.033, H30.039, H30.21, H30.22, H30.23, H30.29, H30.91, H30.92, H30.93, H30.90, H44.011, H44.012, H44.013, H44.019, H44.021, H44.022, H44.023, H44.029, H44.111, H44.112, H44.113, H44.119, H44.131, H44.132, H44.133, H44.139, H43.89, A18.54, B00.51, B02.32, M02.3, L40.50, M45.9,  K52.8 |
| **Lens** | H26.011, H26.012, H26.013, H26.019, H26.031, H26.032, H26.033, H26.039, H26.041, H26.042, H26.043, H26.049, H26.051, H26.052, H26.053, H26.059, H26.131, H26.132, H26.133, H26.139, H26.491, H26.492, H26.493, H26.499, H27.01, H27.02, H27.03, H27.00, H27.111, H27.112, H27.113, H27.119 |
| [**Blepharitis**](https://www.aao.org/Assets/c94319ab-907a-476d-a7dd-0c3025c02810/636713223991730000/bc-2612-icd-10-decisiontrees-blepharitis-mgd-final-v2-pdf?inline=1)**, Eyelid, Orbit, & Meibomian Gland Dysfunction** | H00.011, H00.012, H00.013, H00.014, H00.015, H00.016, H00.019H00.021, H00.022, H00.023, H00.024, H00.025, H00.026, H00.029H00.031, H00.032, H00.033, H00.034, H00.035, H00.036, H00.039, H00.11, H00.12, H00.13, H00.14, H00.15, H00.16, H00.19, H01.001, H01.002, H01.004, H01.005, H01.00A, H01.00B, H01.011, H01.012, H01.013, H01.014 H01.015, H01.016, H01.019, H01.01A, H01.01B, H01.021, H01.022, H01.024, H01.025, H01.02A, H01.02B, H02.051, H02.052, H02.053, H02.054, H02.055, H02.056, H02.059, H02.30, H02.31, H02.32, H02.33, H02.34, H02.35, H02.36, H02.411, H02.412, H02.413, H02.419, H02.421, H02.422, H02.423, H02.429, H02.431, H02.432, H02.433, H02.439, H02.521, H02.522, H02.523, H02.524, H02.525, H02.526, H02.529, H02.821, H02.822, H02.823, H02.824, H02.825, H02.826, H02.829, H02.831, H02.832, H02.833, H02.834, H02.835, H02.836, H02.839, H02.841, H02.842, H02.843, H02.844, H02.845, H02.846, H02.849, H02.881, H02.882, H02.884, H02.885, H02.88A, H02.88B, H05.221, H05.222, H05.223, H05.229, H05.231, H05.232, H05.233, H05.239, H05.241, H05.242, H05.243, H05.249, H05.251, H05.252, H05.253, H05.259, H05.261, H05.262, H05.263, H05.269, H57.811, H57.812, H57.813, H57.819, C43.111, C43.112, C43.121, C43.122, C44.1111, C44.1121, C44.1122, C44.1191, C44.1192, Q10.0, G24.5 |
| **Refractive Errors** | H52.01, H52.02, H52.03, H52.09, H52.11, H52.12, H52.13, H52.19, H52.211, H52.212, H52.213, H52.219, H52.221, H52.222, H52.223, H52.229, Z01.01, Z01.00, Z0.0021, Z01.020, Z21.121, Z21.129 |
| **Ocular Effects in Systemic Diseases** | E10.9, E79.60, E79.62, G70.00, G70.01, M06.0A, M06.0A, M08.00, M08.0A, M08.2A, Q68.0, Q79.61, Q79.62, Q79.69, Q82.5, Q85.01, Q85.09, Q85.8, Q87.11, Q87.42, Q90.9 |
| **Fracture** | S02.30X-, S02.31X-, S02.32X-, S02.40A-, S02.40B-, S02.40C-, S02.40D-, S02.40E-, S02.40F-, S02.121-, S02.122-, S02.129-, S02.400-, S02.401-, S02.403-, S02.831-, S02.832-, S02.839-, S02.841-, S02.842-, S02.849- |
| **Lacrimal** | H04.011, H04.012, H04.013, H04.019, H04.021, H04.022, H04.023, H04.029, H04.111, H04.112, H04.113, H04.119, H04.121, H04.122, H04.123, H04.129, H04.131, H04.132, H04.133, H04.139, H04.521, H04.522, H04.523, H04.529, H04.211, H04.212, H04.213, H04.219, H04.221, H04.222, H04.223, H04.229, H04.541, H04.542, H04.543, H04.549, H04.551, H04.552, H04.553, H04.559, H04.561, H04.562, H04.563, H04.569 |
| **Conjunctivitis** | B30.1, B30.9, H04.121, H04.122, H04.123, H04.129, H10.11, H10.12, H10.13, H11.151, H11.152, H11.153, H11.159, H10.19, H10.211, H10.212, H10.213, H10.219, H10.231, H10.232, H10.233, H10.239, H10.31, H10.32, H10.33, H10.39, H10.401, H10.402, H10.403, H10.409, H10.45, H11.811, H11.812, H11.813, H11.819, H11.821, H11.822, H11.823, H11.829, H18.831, H18.832, H18.833, H18.839, H18.891, H18.892, H18.893, H18.899, M35.00, Y77.11 |
| [**Corneal Dystrophy**](https://www.aao.org/Assets/d2510b11-e02e-47bf-8d98-003542d5b81a/637358923686370000/bc-4606-new-icd-10-decision-trees-corneal-dystrophy-final-pdf) | H18.501, H18.502, H18.503, H18.509, H18.511, H18.512, H18.513, H18.519, H18.521, H18.522, H18.523, H18.529, H18.531, H18.532, H18.533, H18.539, H18.541, H18.542, H18.543, H18.549, H18.551, H18.552, H18.553, H18.559, H18.591, H18.592, H18.593, H18.599 |
| **Corneal Edema/Opacity/**  **Degeneration** | H18.11, H18.12, H18.13, H18.19, H18.211, H18.212, H18.213, H18.219, H18.221, H18.222, H18.223, H18.229, H18.231, H18.232, H18.233, H18.239, H18.331, H18.332, H18.333, H18.339, H18.411, H18.412, H18.413, H18.419, H18.421, H18.422, H18.423, H18.429, H18.441, H18.442, H18.443, H18.449, H18.451, H18.452, H18.453, H18.459, H18.461, H18.462, H18.463, H18.469, H18.611, H18.612, H18.613, H18.619, H18.621, H18.622, H18.623, H18.629, H18.711, H18.712, H18.713, H18.719, H18.731, H18.732, H18.733, H18.739, H17.11, H17.12, H17.13, H17.19, H17.811, H17.812, H17.813, H17.819, H17.821, H17.822, H17.823, H17.829, H17.89 |
| [**Corneal Transplant**](https://www.aao.org/Assets/bce0d6fd-19f9-4830-837c-e08ac1837067/637363430372000000/bc-4606-new-icd-10-decision-trees-corneal-transplant-final-4c-pdf) | S05.01X-, S05.02X-, S05.21X-, S05.22X-, S05.31X-, S05.32X, T86.8401, T86.8402, T86.8403, T86.8411, T86.8412, T86.8413, T86.8421, T86.8422, T86.8423, T86.8481, T86.8482, T86.8483, T86.8491, T86.8492, T86.8493, Z94.7 |
| **Esotropia** | H50.00, H50.011, H50.012, H50.021, H50.022, H50.031, H50.032, H50.041, H50.042, H50.05, H50.06, H50.07, H50.08, H50.312, H50.311, H50.32, H50.43 |
| **Endophthalmitis** | H44.001, H44.002, H44.003, H44.009, H44.011, H44.012, H44.013, H44.019, H44.111, H44.112, H44.113, H44.119, H44.131, H44.132, H44.133, H44.139, H44.19 |
| **Lens** | H26.011, H26.012, H26.013, H26.019, H26.031, H26.032, H26.033, H26.039, H26.041, H26.042, H26.043, H26.049, H26.051, H26.052, H26.053, H26.059, H26.111, H26.112, H26.113, H26.119, H26.131, H26.132, H26.133, H26.139, H26.491, H26.492, H26.493, H26.499, H27.00, H27.01, H27.02, H27.03, H27.111, H27.112, H27.113, H27.119 |
| **Neuro-ophthalmic/ Optic Nerve Disorders** | H46.00, H46.01, H46.011, H46.012, H46.013, H46.019, H46.02, H46.03, H46.10, H46.11, H46.12, H46.13, H46.2, H46.3, H46.8, H46.9, H47.011, H47.012, H47.013, H47.019, H47.091, H47.092, H47.093, H47.099, H47.10, H47.11, H47.20, H47.22, H47.41, H47.42, H47.43, H47.49 |
| **Ectropion** | H02.111, H02.112, H02.114, H02.115, H02.121, H02.122, H02.124, H02.125, H02.131, H02.132, H02.134, H02.135, H02.141, H02.142, H02.144, H02.145, H02.151, H02.152, H02.154, H02.155 |
| **Amblyopia** | H53.011, H53.012, H53.013, H53.019, H53.021, H53.022, H53.023, H53.029, H53.031, H53.032, H53.033, H53.039, H53.041, H53.042, H53.043, H53.049, H53.001, H53.002, H53.003, H53.009 |
| **Hereditary Retinal Dystrophies** | H35.51, H35.52, H35.53, H35.54 |
| **Iridocyclitis** | H20.011, H20.012, H20.013, H20.019, H20.10, H20.11, H20.12, H20.13, H20.20, H20.21, H20.22, H20.23, H20.029, H20.021, H20.022, H20.023, H20.039, H20.031, H20.032, H20.033, H20.049, H20.041, H20.042, H20.043 |
| **Entropion** | H02.011, H02.012, H02.014, H02.015, H02.021, H02.022, H02.024, H02.025, H02.031, H02.032, H02.034, H02.035, H02.041, H02.042, H02044, H02.045, H02.051, H02.052, H02.054, H02.055 |
| **Exotropia** | H50.10, H50.15, H50.16, H50.17, H50.18, H50.111, H50.121, H50.131, H50.141, H50.112, H50.122, H50.132, H50.142, H50.331, H50.332, H50.34 |
| **Neurologic disorders** | G24.5, G35, G43.B0, G43.B1, G43.E01, G43.E09, G43.E11, G43.E19, G45.3, G45.9, G51.31, G51.32, G51.33, G51.39, G70.00, G70.01, G90.2, G93.2, G93.5 |
| **Choroidal Disorders** | D31.30, D31.301, D31.302, D31.303, D31.309, D31.31, D31.32, D31.321, D31.322, D31.323, D31.329, H30.90, H30.91, H30.92, H30.93, H31.011, H31.012, G31.013, G31.019, H31.021, H31.022, H31.023, H31.029, H31.411, H31.412, G31.413, G31.419, H31.421, H31.422, H31.423, H31.429, H59.811, H59.812, H59.813, H59.819 |
| **Viral/Keratitis** | B00.51, B00.52, B00.53, B00.59, B02.31, B02.32, B02.33, B02.34, B02.39, H16.011, H16.012, H16.013, H16.019, H16.021, H16.022, H16.023, H16.029, H16.031, H16.032, H16.033, H16.039, H16.041, H16.042, H16.043, H16.049, H16.051, H16.052, H16.053, H16.059, H16.061, H16.062, H16.063, H16.069, H16.071, H16.072, H16.073, H16.079, H16.121, H16.122, H16.123, H16.129, H16.131, H16.132, H16.133, H16.139, H16.141, H16.142, H16.143, H16.149, H16.211, H16.212, H16.213, H16.219, H16.221, H16.222, H16.223, H16.229, H16.231, H16.232, H16.233, H16.239, H16.251, H16.252, H16.253, H16.259, H16.291, H16.292, H16.293, H16.299, H16.311, H16.312, H16.313, H16.319, H16.321, H16.322, H16.323, H16.329, H16.431, H16.432, H16.433, H16.439, M35.01 |
| **Foreign body/injury/ laceration/mass** | D18.01, D18.09, D31.60, D31.61, D31.62, T26.11XA, T26.11XD, T26.11XS |
| **Retinopathy of Prematurity** | H35.111, H35.112, H35.113, H35.119, H35.121, H35.122, H35.123, H35.129, H35.131, H35.132, H35.133, H35.139, H35.141, H35.142, H35.143, H35.149, H35.151, H35.152, H35.153, H35.159, H35.161, H35.162, H35.163, H35.169, H35.101, H35.102, H35.103, H35.109 |
| **Type I** [**Diabetic Retinopathy**](https://www.aao.org/Assets/8871b228-01b9-4d5f-87c0-c6bbc276ee5b/636063409010130000/bc-708-diabetes-flowchart-cmyk-handout-f-pdf?inline=1) | E10.3211, E10.3212, E10.3213, E10.3219, E10.3311, E10.3312, E10.3313, E10.3319, E10.3411, E10.3412, E10.3413, E10.3419, E10.3511, E10.3512, E10.3513, E10.3519, E10.3521, E10.3522, E10.3523, E10.3529, E10.3541, E10.3542, E10.3543, E10.3549, E10.37X1, E10.37X2, E10.37X3, E10.37X |
|  | E10.3291, E10.3292, E10.3293, E10.3299, E10.3391, E10.3392, E10.3393, E10.3399, E10.3491, E10.3492, E10.3493, E10.3499, E10.3531, E10.3532, E10.3533, E10.3539, E10.3551, E10.3552, E10.3553, E10.3559, E10.3591, E10.3592, E10.3593, E10.3599, E10.9 |
| **Type II** [**Diabetic Retinopathy**](https://www.aao.org/Assets/8871b228-01b9-4d5f-87c0-c6bbc276ee5b/636063409010130000/bc-708-diabetes-flowchart-cmyk-handout-f-pdf?inline=1) | H35.3190, H35.3191, H35.3192, H35.3193, H35.3194, E11.3211, E11.3212, E11.3213, E11.3219, E11.3291, E11.3292, E11.3293, E11.3299, E11.3311, E11.3312, E11.3313, E11.3319, E11.3391, E11.3392, E11.3393, E11.3399, E11.3411, E11.3412, E11.3413, E11.3419, E11.3491, E11.3492, E11.3493, E11.3499, E11.3511, E11.3512, E11.3513, E11.3519, E11.3521, E11.3522, E11.3523, E11.3529, E11.3531, E11.3532, E11.3533, E11.3539, E11.3541, E11.3542, E11.3543, E11.3549, E11.3551, E11.3552, E11.3553, E11.3559, E11.3591, E11.3592, E11.3593, E11.3599 |
|  | H35.341, H35.342, H35.351. H35.352, H35.353, H35.359, H35.361, H35.362, H35.363, H35.369, H35.371, H35.372, H35.373, H35.379, H35.433, H35.439, H35.711, H35.712, H35.713, H35.719, H43.821, H43.822, H43.823, H43.829, H35.3110, H35.3111, H35.3112, H35.3113, H35.3114, H35.3120, H35.3121, H35.3122, H35.3123, H35.3124, H35.3130, H35.3131, H35.3132, H35.3133, H35.3134, H35.3210, H35.3211, H35.3212, H35.3213, H35.3220, H35.3221, H35.3222, H35.3223, H35.3230, H35.3231, H35.3232, H35.3233, H35.3290, H35.3291, H35.3292, H35.3293, H59.031, H59.032, H59.033, H59.039 |
| [**Lagophthalmos**](https://www.aao.org/Assets/2189b610-af6f-4905-8ae9-35787f13f606/636713224682330000/bc-2612-icd-10-decisiontrees-lagophthalmos-final-pdf?inline=1) | H02.201, H02.202, H02.204, H02.205, H02.20A, H02.20B, H02.20C, H02.211, H02.212, H02.214, H02.215, H02.21A, H02.21B, H02.21C, H02.221, H02.222, H02.224, H02.225, H02.22A, H02.22B, H02.22C, H02.231, H02.232, H02.234, H02.235, H02.23A, H02.23B, H02.23C, H02.531, H02.532, H02.534, H02.535 |
| **Glaucoma** | H10.011, H10.012, H10.013, H40.001, H40.002, H40.003, H40.011, H40.012, H40.013, H40.021, H40.022, H40.023, H40.031, H40.032, H40.033, H40.041, H40.042, H40.043, H40.051, H40.052, H40.053, H40.061, H40.062, H40.063, H40.10X2, H40.20X1, H40.111-, H40.112-, H40.113-, H40.121-, H40.122-, H40.123-, H40.131-, H40.132-, H40.133-, H40.141-, H40.142-, H40.143-, H40.151, H40.152, H40.153, H40.211-, H40.212-, H40.213-, H40.221-, H40.222-, H40.223-, H40.231-, H40.232-, H40.233-, H40.241-, H40.242-, H40.243-, H40.31X-, H40.32X-, H40.33X-, H40.31X3, H40.41X-, H40.42X-, H40.43X-, H40.43X2, H40.51X-, H40.52X-, H40.53X-, H40.61X-, H40.62X-, H40.63X-, H40.811, H40.812, H40.813, H40.821, H40.822, H40.823, H40.831, H40.832, H40.833, H44.411, H44.412, H44.413, H44.421, H44.422, H44.423, H44.511, H44.512, H44.513, P15.3, Q15.0,   T49.5X5, T49.5X6, T50.2X5, , T81.31X, T81.83X, T85.390, T85.391, T85.398 |
| [**Myopic Degeneration**](https://www.aao.org/Assets/77438a9d-8936-4275-9f66-0a572a70facf/636395218340500000/e1-1899-med-aaoe-myopicdecisiontree-pdf?inline=1) | H44.2A1, H44.2A2, H44.2A3, H44.2A9, H44.2B1, H44.2B2, H44.2B3, H44.2B9, H44.2C1, H44.2C2, H44.2C3, H44.2C9, H44.2D1, H44.2D2, H44.2D3, H44.2D9, H44.2E1, H44.2E2, H44.2E3, H44.2E9, H44.21, H44.22, H44.23 |
| **Vitreous Body Degeneration** | H43.10, H43.11, H43.12, H43.13, H43.20, H43.21, H43.22, H43.23, H43.391, H43.392, H43.393, H43.399, H43.811, H43.812, H43.813, H43.819, H43.821, H43.822, H43.823, H43.829, H43.89 |
| [**Panuveitis**](https://www.aao.org/Assets/c74704c3-6cee-4d67-b4fd-d9ac59057974/637572246475500000/panuveitis-icd10-diagram-bc4810-20211001-pdf) | H44.00-, H44.11-, H44.12-, H44.13-, H44.02-, H20.03-, H20.04- |
| **Palsies** | H49.00, H49.01, H49.02, H49.03, H49.10, H49.11, H49.12, H49.13, H49.20, H49.21, H49.22, H49.23, H49.30, H49.31, H49.32, H49.33, H49.40, H49.41, H49.42, H49.43, H49.881, H49.882, H49.883, H49.889, H49.9 |
| **Pterygium** | H11.021, H11.022, H11.023, H11.029, H11.031, H11.032, H11.033, H11.039, H11.041, H11.042, H11.043, H11.049, H11.051, H11.052, H11.053, H11.059, H11.061, H11.062, H11.063, H11.069 |
| **Nystagmus** | H55.00, H55.01, H55.02, H55.03, H55.04, H55.09, H55.81, H55.89 |
| **Strabismus** | H44.22A1, H44.22A2, H44.22A3, H44.22A9, H44.22B1, H44.22B2, H44.22B3, H44.22B9, H44.22C1, H44.22C2, H44.22C3, H44.22C9, H44.22D1, H44.22D2 H44.22D3, H44.22D9, H44.22E1, H44.22E2, H44.22E3, H44.22E9, H50.21, H50.22, H50.50, H50.51, H50.52, H50.53, H50.54, H50.55, H50.60, H50.611, H50.612, H50.69, H50.811, H50.812, H50.89, H50.9, Q10.3 |
| **Vertigo** | H02.411, H02.412, H02.413, H02.419, H02.421, H02.422, H02.423, H02.429, H02.431, H02.432, H02.433, H02.439, Q10.0 |
| **Visual Disturbances** | H53.021, H53.022, H53.023, H53.029, H53.031, H53.032, H53.033, H53.039, H53.19, H53.2, H53.40, H53.47, H53.421, H53.422, H53.423, H53.429, H53.461, H53.462, H53.469, H53.52 |
| **Vision Rehabilitation** | H54.0X33, H54.0X34, H54.0X35, H54.0X43, H54.0X44, H54.0X45, H54.0X53, H54.0X54, H54.0X55, H54.1131, H54.1132, H54.1131, H54.1141, H54.1142, H54.1151, H54.1152, H54.1213, H54.1214, H54.1215, H54.1223, H54.1224, H54.1225, H54.2X11, H54.2X21, H54.2X12, H54.2X22, H54.413A, H54.414A, H54.415A,H 54.42A3, H54.42A4, H54.42A5, H54.511A, H54.512A, H54.52A1, H54.52A2 |
| **Vitreous Body Degeneration** | H43.11, H43.12, H43.13, H43.10, H43.21, H43.22, H43.23, H43.20, H43.391, H43.392, H43.393, H43.399, H43.811, H43.812, H43.813, H43.819, H43.821, H43.822, H43.823, H43.829 |
| **Peripheral Retinal Degeneration** | H35.411, H35.412, H35.413, H35.419, H35.421, H35.422, H35.423, H35.429, H35.431, H35.432, H35.433, H35.439, H35.441, H35.442, H35.443, H35.449, H35.461, H35.462, H35.463, H35.469 |
| **Retinal Detachments** | H33.011, H33.012, H33.013, H33.019, H33.101, H33.102, H33.103, H33.109, H33.191, H33.192, H33.193, H33.199, H33.031, H33.032, H33.033, H33.039, H33.041, H33.042, H33.043, H33.049, H33.051, H33.052, H33.053, H33.059, H33.21, H33.22, H33.23, H33.20, H33.41, H33.42, H33.43, H33.49, H33.8 |
| **Retinal Tears** | H33.301, H33.302, H33.303, H33.309, H33.311, H33.312, H33.313, H33.319, H33.321, H33.322, H33.323, H33.329, H33.331, H33.332, H33.333, H33.33 |
| **Retinal Vascular Occlusion** | H34.10, H34.11, H34.12, H34.13, H34.8110, H34.8120, H34.8130 H34.8190, H34.8111, H34.8121, H34.8131, H34.8191, H34.8112, H34.8122, H34.8132, H34.8192, H34.8310, H34.8320, H34.8330, H34.8390, H34.8311, H34.8321, H34.8331, H34.8391, H34.8312, H34.8322, H34.8332, H34.8392 |
| **Separation of Retinal Layers** | H35.711, H35.712, H35.713, H35.719, H35.721, H35.722, H35.723, H35.729, H35.731, H35.732, H35.733, H35.739 |
| **Other Retinal Disorders** | H35.021, H35.022, H35.023, H35.029, H35.031, H35.032, H35.033, H35.039, H35.041, H35.042, H35.043, H35.049, H35.051, H35.052, H35.053, H35.059, H35.20, H35.21, H35.22, H35.23, H35.60, H35.61, H35.62, H35.63, H35.81, H35.82 |
| **Miscellaneous** | C69.20, C69.21, C69.22, D31.60, D31.61, D31.62, D32.0, D35.2, E05.00, E05.01, F08.71, F44.679, G24.5, G43.B0, G43.B1, G43.101, G43.109, G43.111, G43.119, G43.801, G43.809, G43.811, G43.819, G45.3, G45.9, G51.31, G51.32, G51.33, G51.39, G80.9, H02.89, H05.111, H05.112, H05.113, H05.119, H30.011, H30.012, H30.012, H30.019, H34.211, H34.212, H34.213, H34.219, H44.521, H44.522, H44.523, H44.529, H47.611, H47.612, H49.41, H49.42, H49.43, H49.40, H51.11, H51.21, H51.22, H51.23, H51.20, H51.8, H53.131, H53.132, H53.133, H53.1139, H53.121, H53.122, H53.123, H53.129, H53.19, H53.2, H53.8, H54.7, H57.051, H57.052, H57.053, H57.059, H57.11, H57.12, H57.13, H57.10, H57.89, H57.02, H57.9, H59.021, H59.022, H59.023, H59.029, I63.9, L57.0, L82.1, L93.0, M06.9, P04.40, Q07.8, Q13.1, R51.0, R51.9, R48.3, R70.0, R73.03, R73.09, S00.11X-, S00.12X-, S05.01X-, S05.00X-, S05.02X-, T15.01X-, T15.02X-, T15.10X-, T15.11X-, T15.12X-, T15.81X-, T15.82X-, T26.00X-, T26.01X-, T26.02X-, Z05.8, Z79.899 |
| [**Age-Related Macular Degeneration**](https://www.aao.org/Assets/1b80d71c-50cb-4c54-880d-7053ebf44774/636063406837370000/bc-708-armd-decisiontree-cmyk-handout-f-pdf?inline=1) | H35.3111, H35.3112, H35.3113, H35.3121, H35.3122, H35.3123, H35.3131, H35.3132, H35.3133, H35.3211, H35.3212, H35.3213, H35.3221, H35.3222, H35.3223, H35.3231, H35.3232, H35.3233 |

**Table S1.** Ophthalmic ICD-10 diagnostic codes used to identify patients in this study. ICD: International Classification of Diseases.

| **Administrative Concerns** | **Scheduling/ canceling** | Patients coordinate ophthalmology visits—scheduling, rescheduling, cancellations, and referrals for in-person or video consults—and need clear communication with schedulers, technicians, and providers for timely care. |
| --- | --- | --- |
|  | **Pharmacy/**  **Medication Refill** | Patients struggle to refill prednisone/prednisolone at retail pharmacies, handling authorizations, pickups, and bottle exchanges while seeking relief from persistent swelling. |
|  | **Insurance** | Patients struggle with insurance authorizations—denials, Medicare navigation, and appeals—and billing processes, causing financial stress and delaying essential ophthalmic care. |
|  | **Disability** | Patients with vision-limiting eye disease apply for SSDI and FMLA, burdened by complex paperwork—completing, submitting, and mailing forms—that delays their benefit access. |
|  | **Vision** | Patients seek updated glasses or contact lens prescriptions for blurred, tilted, or floating vision, undergo stereotests and refraction, and have retinopathy, macular changes, or keratoconus, prompting LASIK discussion. |
|  | **Glaucoma** | Glaucoma and ocular hypertension patients use topical/systemic pressure‐lowering agents (timolol, dorzolamide, latanoprost, Lumigan, Zioptan, Diamox), request renewals, schedule IOP checks, and manage discomfort with OTC analgesics. |
|  | **Tumor** | Patients undergo imaging (with/without contrast), blood tests, biopsies, and genetic analysis to diagnose ocular/orbital lesions, then receive targeted treatments like laser procedures. |
|  | **Surgery** | Patients undergoing ocular procedures (Botox, strabismus surgery, blepharoplasty) focus on perioperative care—anesthesia, incision management, steroids, antibiotics, antihistamines—and address swelling, bleeding, infection, itching, and tear support. |
|  | **Complications** | Patients with myasthenia gravis (Mestinon), thyroid orbitopathy (Tepezza), or autoimmune disease (Plaquenil) report vision disturbances requiring neurologic, rheumatologic, and endocrine coordination, invasive procedures, and immunosuppression management. |
|  | **Swelling** | Patients experience eyelid/eyelash swelling (styes/infections) and conjunctivitis managed with drops; some seek surgical eyelid/brow incisions for persistent lesions and schedule acute eye-care appointments. |
|  | **Cornea**  **Clinical Concerns** | Patients with cataracts schedule lens-replacement to treat vision loss, coordinating preoperative assessments, surgery logistics, and postoperative care for restored vision. |

**Table S2.** Ophthalmic message topics classified by administrative versus clinical issues. IOP: intraocular pressure; FMLA: Family and Medical Leave Act; SSDI: Social Security Disability Insurance.

| **Pre-defined Message Topics** | | **N** | **%** |
| --- | --- | --- | --- |
| **Administrative**  **Concerns** | Scheduling/canceling | 4,166 | 29.2 |
|  | Pharmacy/medication refill | 1,352 | 9.5 |
|  | Insurance | 581 | 4.1 |
|  | Disability | 221 | 1.5 |
|  | **Total Administrative Concerns** | **6,320** | **44.2** |
| **Clinical**  **Concerns** | Vision | 2,975 | 20.8 |
|  | Glaucoma | 1,241 | 8.7 |
|  | Tumor | 1,074 | 7.5 |
|  | Surgery | 1,064 | 7.4 |
|  | Complications/other conditions^a^ | 774 | 5.4 |
|  | Swelling/infection | 525 | 3.7 |
|  | Cornea | 311 | 2.2 |
|  | **Total Clinical Concerns** | **7,964** | **55.8** |
| **Total Pre-defined Messages** | | **14,284** | **100.0** |

**Table S3.** Messages categorization based on pre-definition. ^a^Complications/other conditions: other conditions or referrals not covered by the list in the current table including; pneumonia, myasthenia gravis, vertigo, or thyroid issues.

**Figure S1.** Workflow of patient message data extraction, processing, and analysis.
